# Analysis of the Current and Future Prediction of Land Use/Land Cover Change Using Remote Sensing and the CA-Markov Model in Majang Forest Biosphere Reserves of Gambella, Southwestern Ethiopia

**DOI:** 10.1155/2021/6685045

**Published:** 2021-02-23

**Authors:** Semegnew Tadese, Teshome Soromessa, Tesefaye Bekele

**Affiliations:** ^1^Addis Ababa University, Center of Environmental Sciences, Addis Ababa, Ethiopia; ^2^Ethiopian Environments and Forestry Research Institute, Addis Ababa, Ethiopia

## Abstract

This study aimed to evaluate land use/land cover changes (1987–2017), prediction (2032–2047), and identify the drivers of Majang Forest Biosphere Reserves. Landsat image (TM, ETM+, and OLI-TIRS) and socioeconomy data were used for the LU/LC analysis and its drivers of change. The supervised classification was also employed to classify LU/LC. The CA-Markov model was used to predict future LU/LC change using IDRISI software. Data were collected from 240 households from eight kebeles in two districts to identify LU/LC change drivers. Five LU/LC classes were identified: forestland, farmland, grassland, settlement, and waterbody. Farmland and settlement increased by 17.4% and 3.4%, respectively; while, forestland and grassland were reduced by 77.8% and 1.4%, respectively, from 1987 to 2017. The predicted results indicated that farmland and settlement increased by 26.3% and 6.4%, respectively, while forestland and grassland decreased by 66.5% and 0.8%, respectively, from 2032 to 2047. Eventually, agricultural expansion, population growth, shifting cultivation, fuel wood extraction, and fire risk were identified as the main drivers of LU/LC change. Generally, substantial LU/LC changes were observed and will continue in the future. Hence, land use plan should be proposed to sustain resource of Majang Forest Biosphere Reserves, and local communities' livelihood improvement strategies are required to halt land conversion.

## 1. Introduction

Land cover and land use represent the assimilating elements of the resource base. Land use describes activities, arrangements, and inputs often associated with people that take place on the land and represent the current use of property such as residential homes, shopping centres, row crops, tree nurseries, state parks, and reservoirs. Land cover describes the natural and anthropogenic features that can be observed on the Earth's surface, i.e., forests, tidal wetlands, developed/built areas, grasslands, and water [1–3]. Land use/land cover (LULC) change is perhaps the most important concern in many regions of the world [4–7]. It is recognized that dramatic LULC change can significantly impact regional climate, ecosystem stability, water balance, stream silt up, socioeconomic practices, and biodiversity [8–14]. As the pressure of the LULC change is increasing in many places, understandings of current and future LULC changes and patterns are a critical issue and seek timely analysis [8, 9, 15]. LU/LC change is significantly increasing and primarily activated by natural phenomena and anthropogenic activities [10, 11]. To collect information and time serious LU/LU change, ground surveys and satellite sensors can be utilized [12].

Prediction of LU/LC using time serious data is important for the future management plan of LULC [13], and it is regularly employed for a diverse suitability measure as a proxy of human influence on land change processes. A Markov model is one in which the future state of a system can be predicted purely based on the proximately preceding state. Predicting future change is achieved by creating a transition probability matrix of LULC change from period one to period two [14]. Multispectral satellite images and the CA-Markov chain model were used by the researcher to predict the LULC change in different regions [16–18]. It also computed states between different land uses and quantified the transition rate between different land uses [19]. The factors in which driving forces of LULC change were combined to provide the estimation of future scenarios [20].

In Ethiopia, LULC changes are a persistent event where agricultural activities and settlements are dominated in the rural landscapes. Recent studies indicated that land use/land cover change is increasing; predominantly, expansion of agricultural land at the expense of natural forest was observed in different parts of Ethiopia [8, 13, 15, 21–31]. For instance, Kindu et al. [8] reported that about 66.2% of woodland converted to farmland in Munessa-Shashemene of Oromia, Ethiopia. However, nearly 27% increase of forest cover was gained as results of community afforestation and rehabilitation activities on degraded hilly lands in Chemoga watershed within the Blue Nile, Ethiopia [32, 33].

Moreover, LULC changes analysis has been conducted to identify driving forces of the changes in different parts of Ethiopia [13, 27, 34–37]. For example, a study from Afar region identified more than fifteen LULC changes driving factors such as migration triggered by drought, land tenure, and government policy changes [34]. The study from the central rift valley revealed that population growth, a decline in agricultural productivity, land tenure change, and erratic rainfall are the major drivers of LULC changes in the study area [37]. Likewise, driving factors of LULC changes are diverse in different places or regions. Hence, driving factors of LULC changes of certain ecosystems or places should be addressed and investigated locally based on the agroecology and socioeconomic condition of the area.

Analysis and prediction of LULC changes have significant roles in the understanding of earth-atmosphere interaction, forest fragmentation, biodiversity loss, and future management plans [9, 38–41]. Also, inspection and analysis of LULC have greatly increased in providing the most accurate evaluation of the world's forest, grassland, and agricultural resources regarding their spread status and health [42]. However, studies concerning LULC changes, drivers, and prediction have not been performed in Majang Zone, a place where the UNSECO registered forest biosphere reserve was established recently. The objectives of this study are to analyse LU/LC changes over the last three decades 1987–2017, predict future LU/LC change from 2017 to 2047, and identify LU/LC changes drivers of the Majang Forest Biosphere Reserve (MFBR).

## 2. Materials and Methods

### 2.1. Description of the Study Area

**Figure 1:**
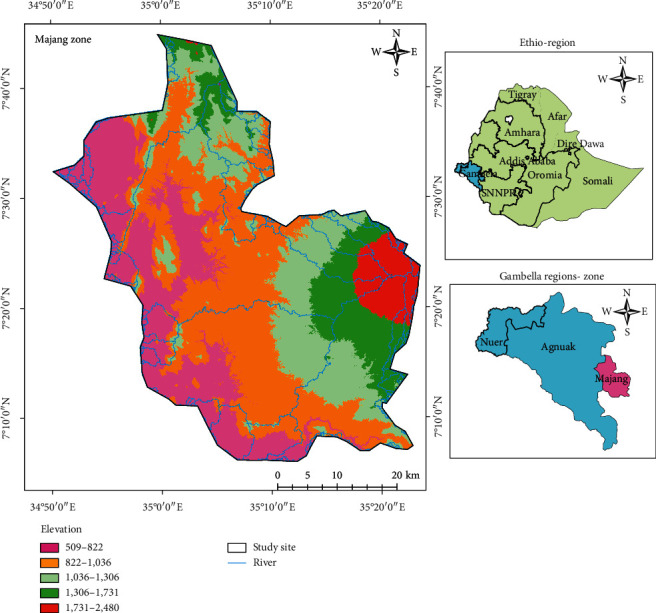
Location of the study area.

This study was conducted in Majang Forest Biosphere Reserve (MFBR) which is found in the Majang Zone, Gambella National Regional State of Ethiopia. It is unique biogeography and shares a border with Illubabor Zone of Oromia Regional State and Sheka and Bench-Maji zones of the Southern Nations, Nationalities, and People (SNNP). It covers a total area of 233,254 ha of forest, woodland, agricultural land, rural settlement, and towns (Figure 1). MFBR is located between 07°08′-07°23′ latitude and 035°04′-035°19′ longitude, and the area has an altitude range of 562–2444 m [1].

**Figure 2:**
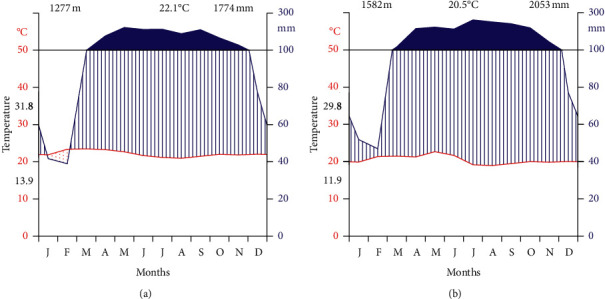
Mean annual temperature and rainfall recorded in (a) Tinishu Meti (1987–2017) and (b) Ermichi (1987–2017) Metrological stations.

The climate of the zone is generally characterized by a hot and humid type, which is marked on most rainfall maps of Ethiopia as being the wettest part of the country (Figure 2). The annual average rainfall is 1774, and means annual minimum and maximum monthly temperature ranges between 13.9°C and 31.8°C in Tinishu Meti Metrological Station (Figure 2(a)). The annual average rainfall is 2053, and mean annual minimum and maximum monthly temperature ranges between 11.8°C and 29.7°C in Ermichi Metrological Station (Figure 2(b)). The maximum average monthly temperature is in February (29.8°C and 31.8°C) while the minimum is in January (11.9°C and 13.9°C), in Ermichi and Tinishu Meti, respectively. The maximum rainfall is between April and October and low rainfall from November to March (NMSA, 2019) (Figure 2).

The major vegetation types of the forest biosphere reserves are Montana evergreen forest, lowland semievergreen forest, and riparian vegetation (WBISPP, 2000). Besides, the vegetation of this area has different categories in terms of life forms such as a high natural forest, woodlands, bushlands, and grasslands which are the major vegetation types in the forest biosphere reserves, and it is categorized under moist Afromontane forest among four Ethiopian biome categories (MEFCC, 2017).

### 2.2. Data Acquisition and Processing

Freely available satellite imagery (Landsat-5 TM (1987), Landsat-7 ETM+ (2002), and Landsat-8 OLI-TIRS (2017)) was downloaded from the USGS website https://earthexplorer.usgs.gov/ of Earth explorer. These data were projected to Universal Transverse Mercator (UTM) with a datum of the World Geodetic System 84 (WGS84), projection system zone 36 N (Table 1). Dataset selection was fixed in the dry season when a clear sky period occurs in which the lowest or zero monthly cloud cover is achieved.

Atmospheric correction (FLAASH module), geometric correction, mosaicking, and masking were performed during preprocessing using ENVI version 5.3 before classifying the images [35, 36]. Atmospheric correction and geometric correction were used to avoid sensor noise, haze, adjustment of data loss, and missing line because of solar position and satellite calibration [43].

Prior to image classifications, ground reference points (GRP) were collected through direct field observation to verify classified images with land use/cover type. The total numbers of GRP collected in the study area were 250, with 50 GRP for each LU/LC type using a global positioning system (GPS). Pixel-based supervised image classification with the maximum likelihood classification (MLC) algorithm [19, 20, 37] was carried out for image classification of the study periods. In supervised classification, region of interest (ROI) was applied as a signature for each land use class. A total of 300 ROI signatory was made with the maximum number of ROI in forestland (90) and the minimum number in waterbody (30).

Based on the existing feature of LULC in the study area landscape, the Coordination of Information on the Environment (CORINE) LULC classification system was used to classify the LULC classes [44]. Therefore, we classified LULC into five classes: forestland, farmland, grassland, settlement, and waterbody. The LULC classes together with their description are presented in Table 2. ENVI 5.3 and Arc GIS 10.4.1 softwares were used to image classification.

The accuracy assessments were accomplished for classified images of 1987, 2002, and 2017 by applying a minimum of 40 random points created as per class with stratified random sampling, for which the corresponding reference classes of each LULC class were collected by field visit [46, 47]. Then, the accuracy assessments were computed using a confusion matrix (ground truth ROI). Therefore, the results of the accuracy assessment of the classified image showed an accuracy of 85.5%, 86%, and 87.3%, and Kappa statistics were in the range of 0.81–0.83% for the years 1987, 2002, and 2017, respectively (Table 3).

### 2.3. Suitability LU/LC and Input Data Preparation

The classified maps were reclassified considering the priority of suitability for each LULC class, and each reclassifies map was weighted and overlayed by including factors such as distance to road, slope, and altitude in Arc GIS. The constraints are a condition that limits the expansion of LULC classes. The factors give a degree of suitability for an area to be changed. The constraints and factors considered were distance to road, elevation, and slope suitable areas for conversion to each class. The constraints are articulated in the Boolean maps where the suitable areas were set a value of 1, while the area not suitable was set a value of 0 [48]. The factors were changed to binary format from 0 to 255, in which 255 is highly suitable and 0 is not suitable using IDRISI version 17.0 for further processing.

**Figure 3:**
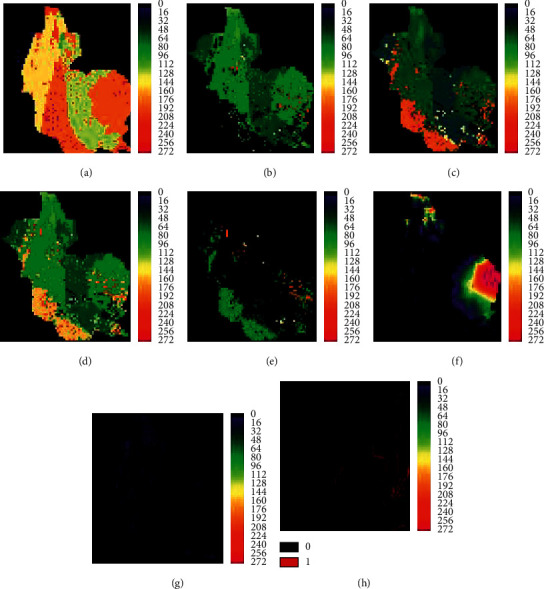
The suitability maps and input data: forestland (a), farmland (b), grassland (c), settlement (d), and waterbody (e) are suitability maps. Elevation (f), slope (g), and road (h) are the input data.

The suitability maps (forestland, farmland, grassland, settlement, and waterbody) were derived using the Decision Wizard module in IDRISI (Figure 3). First, the constraints were standardized into Boolean maps, and then, the fuzzy function combined with the weighted linear combination (WLC) was used to process the standard factors. The factors were stretched from 0 to 255 with fuzzy function (sigmoidal), by monotonically increased control point. The weights of the factors resulted from the AHP function in the WLC module. Then, the transition suitability maps of all classes were made in the MCE module using the constraints, factors, and weights. Finally, the suitability and factors images were used as an input in the Markov change prediction model.

### 2.4. Simulation of LULC Change Using the CA-Markov Chain Model (CA-MCM)

A CA-Markov model is applied to use both special and temporal LULC changes modelling [49, 50]. The CA-Markov model combines cellular automata and Markov chain to predict the characteristics and trends of LULC change over time, provide a better understanding of the factors that drive forest changes, and generate future land use/land cover scenarios to support the design of policy responses [22, 51, 52]. Moreover, this model is commonly used to illustrate the dynamics of LULC, forest cover, settlement expansion, plant growth, and modelling of watershed management. It is also significant to land use policy design and planning for sustainable land use development [53]. Therefore, it is essential to study the chronological LULCC to understand the relations between humans and the environment from a long-term view [51].

**Figure 4:**
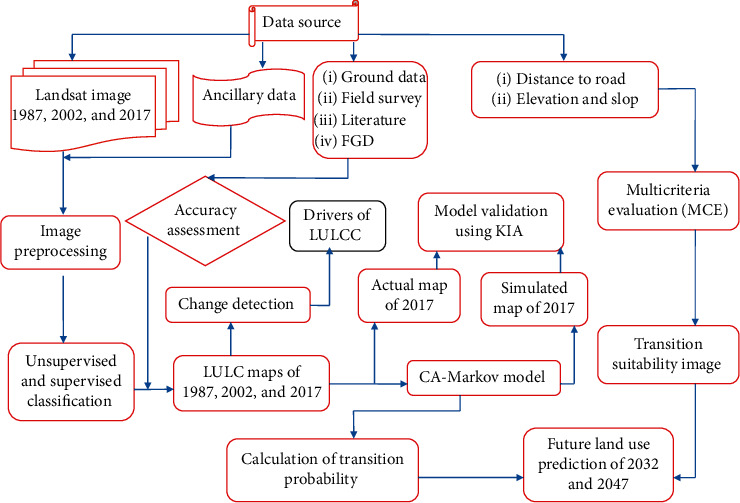
A schematic representation of the study adopted from Yirsawet al. [17].

To predict future LULC changes for the study site, IDRISI software version 17 (CA-Markov model) was used. While doing so, the following specific processes were followed: (a) LU/LC maps for the years 1987 and 2017 were used to obtain the transition probabilities image [52, 54]. (b) Considering the CA-Markov model approach, the LULC for the year 2017 was simulated using the transition probabilities of the year 1987–2002. (c) The transition suitability image was computed using constraints and factors in the multicriteria evaluation (MCE) module [55–57]. (d) Finally, the LULC for the year 2032 and 2047 were projected using the transition probabilities images, base map, and transition suitability image (Figure 4).

#### 2.4.1. CA-Markov Chain Model (CA-MCM) Approach

The CA-Markov model is the integration of cellular automata and transition probability matrix created by the cross-tabulation of two dissimilar images [58]. This integration of the CA-Markov model offers a strong method in spatial and temporal dynamic modelling [58, 59]. In other words, the CA-Markov chain can simulate and predict any transitions among any number of categories [60, 61]. CA is a dynamic procedure model that is frequently used in a spatial model for predicting future land use/land cover change [61–63]. The important properties of CA are that they show the spatial and dynamic process and that is why they have been broadly used in land use/land cover simulation [61]. The CA model is shown in the following equation (1) [53, 64, 65].

(1) $$\begin{array}{c} S\;\left(t,\;t+1\right)=f\left(S\left(t\right),\;N\right), \end{array}$$

where *S* (*t* *+* 1) is the system status at the time of (*t, t* *+* 1), functioned by the state probability of any time (*N*).

The Markov chain model is often used in LULC monitoring, ecological modelling, simulation changes, trends of the LULC, and to predict the extent of the land use change and the stability of future land development in the area of concern [50, 62, 65]. The Markov chain model pronounces the LULC change from one time to another to predict future change [66, 67]. Equation (1) explains the calculation of the prediction of LULC changes (Markov chain model):

(2) $$\begin{array}{c} S\left(t,t+1\right)=P\text{ij}\times S\left(t\right), \end{array}$$

where *S* (*t*) is the system status at the time of *t*, *S* (*t* *+* 1) is the system status at the time of *t* *+* 1; *P*ij is the transition probability matrix in a state which is calculated as follows [19, 66]:

(3) $$\begin{array}{c} =\;\left\|P_{\text{ij}}\right\|=\;\left\|\begin{array}{ccc} P1,1 & P1,2 & P1,N\\ P1,1 & P2,2 & P2,N\\ \cdots & \cdots & \cdots \\ \text{PN},1 & \text{PN},2 & \text{PN},N \end{array}\right\|, \end{array}$$

(4) $$\begin{array}{c} \left(0\;\leq P_{\text{ij}}\leq 1\right), \end{array}$$

where *P* is the transition probability; *P*_ij_ stands for the probability of transforming from present state *i* to another state *j* in succeeding time; PN is the state probability of any time. The high transition has probabilities near (1) and the low transition will have a probability near (0) [66]. Markov Chain concludes precisely how much land would be estimated to change from the latest date to the predicted date. The transition probabilities file is the result of this process, which is a matrix that registers the probability that each land use/land cover class will change to every other class [68].

#### 2.4.2. CA-Markov Model Validation Approach

The use of kappa indexes for the calculation determines the overall achievement rate, and it delivers an understanding of the real factors in the strength or weakness of the results. When 75% ≤Kappa <100, the result maps are in a high level of agreement; if 50% ≤Kappa ≤75%, the result maps are in a medium level of agreement; and if Kappa ≤50, the result maps are in a poor agreement [69, 70]. Therefore, to know the accuracy of the CA-Markov model in simulating future LULC conditions, the model was confirmed [55] after simulating the 2017 LULC situations using the 1987 and 2002 classified images. Kappa index of agreement (*K*_IA_) [71] such as Kappa for no information (*K*_no_), Kappa for location (*K*_location_), and Kappa for standard (*K*_standard_) [55, 71] evaluated the agreements of the two maps (actual and simulated 2017) using the CROSSTAB Module in IDRISI. Besides, comparisons of the simulated and the actual area of each LULC class were also performed using the validate module. Hence, the kappa index is acceptable; the land use and land cover in 2032 and 2047 can be predicted. The following equations express the statistics for the kappa variations according to Omar et al. [55]:

(5) $$\begin{array}{c} K_{\text{no}}\;=\frac{\left(M\left(m\right)N\left(n\right)\right)}{P\left(p\right)-N\left(n\right)},\\ K_{\text{location }}=\;\frac{\left(M\left(m\right)N\left(n\right)\right)}{P\left(p\right)-N\left(n\right)},\\ K_{\text{standard}}\;=\frac{\left(M\left(m\right)N\left(n\right)\right)}{P\left(p\right)-N\left(n\right)}, \end{array}$$

where no information is defined by *N* (*n*), medium grid cell-level information by *M* (*m*), and perfect grid cell-level information across the landscape by *P* (*p*).

#### 2.4.3. Land Use/Land Cover Change Analysis

Following the classification of images (1987, 2002, and 2017) and prediction of the 2032 and 2047 situation, change statistics were computed through comparisons among the successive periods [13, 47, 71–74]. The conversion matrix of the past 30 years periods (1989–2017) was made to differentiate the changes of each land-use type [25, 34, 46, 72]. Furthermore, the percentage of change and rate of change was also computed using equations (3) and (4), respectively [13, 29, 72, 75].

(6) $$\begin{array}{c} \text{RC }\left(\frac{\text{ha}}{\text{year}}\right)=\left(\frac{X-Y}{Z}\right),\\ \%\text{ of change}=\left(\frac{X-Y}{Y}\right)*100, \end{array}$$

where RC is the rate of change, *X* is the area of LULC (ha) in the recent year, *Y* is the area of LULC (ha) in the past year, and *Z* is the time interval between *X* and *Y* in years.

### 2.5. Source of Data for LULC Change Drivers

A field survey was conducted to explore the socioeconomic data for showing the driver of LU/LC change based on the survey qualitative tools such as focus group discussion (FGD), key informant interview (KII), and household (HH) survey including field observation and document review. The ultimate purpose of the field survey was to collect quantitative data to help better understand, explain, and interpret the LULC change drivers [76–79] using semistructured questionnaires (close and open-ended). The data were generated from both primary and secondary sources. To do so, eight kebeles were purposively selected from two districts, Godere and Mengeshi, namely, Ashani, Baya, Fejeji, Newe, Akashi, Dunchaye, Gonchi, and Gelesha.

Household (HH) survey was conducted in eight kebeles of Godere and Mengeshi districts (four kebeles per district) from 10 March to May 2019. These kebeles were selected, based on the level of forest resources dependency communities. So, a total of 240 HHs were randomly selected and interviewed [80]. The questionnaires were envisioned to capture drivers of LULC changes perception, socioeconomic features of HHs, and related information [27]. Furthermore, FGD (head of agricultural office, natural resource expert, elders, women, model farmers, kebeles administrative chairman, and representatives of NGO working in the woredas) and KII (elders, leaders, and women) were conducted in all the selected kebeles for detailed analyses of LULC change drivers (Table 4). During the interviews and discussion, the main attentions were to get adequate information about the past and present trends of LULC change and identify the main driving causes of LULC change. The farmers were asked to explain what parts of the landscape were changed, describe the consequences of the changes in their livelihood, surroundings, and environment, and how their socioeconomic activity contributes to the land-use change.

#### 2.5.1. Data Analysis of Household Survey

All the collected data from the respondents were subjected to descriptive statistics using SPPS version 20 software. Averages, percentages, and frequency descriptive statistics were used to describe HHs' socioeconomic characteristics, a ranking of LULC change drivers along with presenting in tables and graphs.

## 3. Results

### 3.1. Status of Land Use/Land Covers

**Figure 5:**
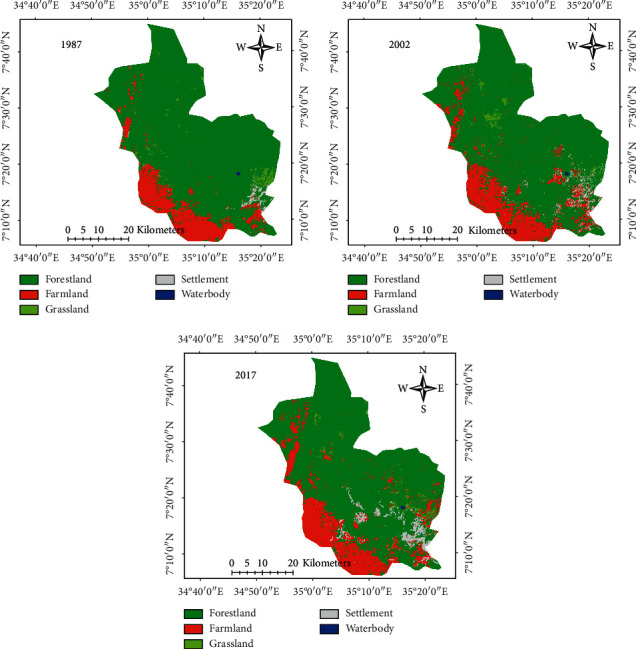
LULC of 1987, 2002, and 2017 in MFBR.

Five LULC classes were identified in the study area for the specified period 1987–2017 (Figure 5). In 1987, the forestland accounts for 84.4% followed by farmland (13.2%), grassland (1.5%), and settlement and waterbody that cover 0.9% and 0.06% which shows minimal coverage of the Majang Forest Biosphere Reserve (Table 5). In 2002, forestland cover about 80.8% is followed by farmland (15.8%), while settlement, grassland, and waterbody accounted for 2%, 1.3%, and 0.06%, respectively. Forestland was declined to 77.8% in 2017, whereas farmland, settlement, and grassland increased by 17.4%, 3.4%, and 1.4%, respectively, but waterbody showed no significant changes with that of 1987 and 2002 (Table 5).

In all study periods, farmland and settlement significantly increase as the expense of forestland and grassland coverage, in which the forestland and grassland were decreased by 19,6761.6 to 18,1504.9 and 3,509.2 to 3,192.2 from 1987 to 2017 (Table 5).

### 3.2. LULC Conversions Analysis: 1987–2002 and 2002–2017

The land use/land cover change was performed by taking the initial year in 1985. Three LULC conversions were detected, i.e., between 1987 and 2002, 2002 and 2017, and 1987 and 2017. The land use/land cover conversions results revealed that a substantial loss and gains of LULC were inspected in the first (1987–2002), second (2002–2017), and third study periods. For instance, forestland was converted to other LULC classes during the first, second, and third study periods by about 556.5 ha (4.4%), 460.6 ha (3.8%), and 1017.1 ha (8.4%), respectively. Also, grassland was reduced by about 28.6 ha (13.6%) and 21.1 ha (9.5%) in the first and third study periods, respectively, while it increases by 7.5 ha 3.6% in the third study period. On the contrary, farmlands and settlements were expanded by 408.3 ha (16.6%), 243.2 ha (9%), and 651.5 ha (24.1%) and 179.6 ha (56.7%), 208.1 ha (39.8%), and 387.7 ha (73.9%) in the first, second, and third study periods, respectively, while in waterbody, only 0.3 ha converts to other land use in all study periods (Table 6).

The conversions of LU/LC from one class to another class were revealed in all study periods (Tables 7 and 8). The diagonals in the matrix from the tables are the persistence, while the off-diagonals are the conversions from one category to the others. Between 1987 and 2002 periods, 3732, 510, and 47 ha of farmland were converted from forestland, settlement, and grassland, respectively, while farmland gained from other LU/LC categories (Table 7). During this period, some areas of settlement were also converted from farmland (519 ha), forestland (458 ha), and grassland (10 ha). Although, about 2764 ha, 510 ha, and 402 ha of the settlement were also converted to forestland, farmland, and grassland, respectively. Gains and losses in forestland and grassland were also taken place in all study periods (Table 7).

Between, 2002 and 2017 periods, 4312 ha, 1773 ha, 1114 ha, and 6 ha of forestland were also converted to farmland, grassland, settlement, and waterbody, respectively. Similarly, forestland, grassland, and settlement were also gained from other LU/LC categories (Table 8). In these periods, a significant area of farmland was converted from forestland (4312 ha), settlement (2418), and grassland (206 ha). In reverse, there was also a considerable conversion of farmland to other categories. A significant amount of gains and losses in the settlement has also occurred in these periods (Table 8).

### 3.3. Future Land Use/Land Cover Change

#### 3.3.1. Actual and Simulated LULC of MFBR for 2017

**Figure 6:**
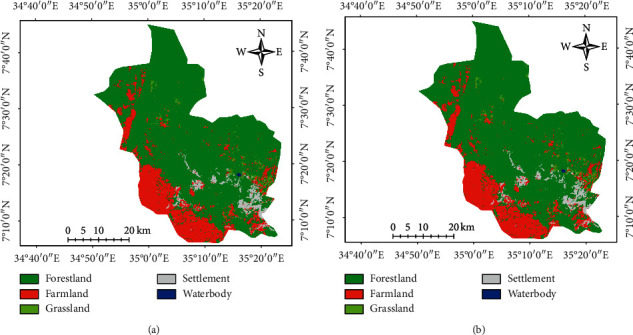
The actual (a) and simulated (b) LULC maps of MFBR for 2017.

Actual and simulated LULC of MFBR was developed for the year 2017. Accordingly, the actual and simulated maps of the year 2017 depicted soundly similarity in waterbody cover, while slight differences were depicted in other LULC classes (Figure 6). The area coverage of the two maps showed that all land use/land cover classes have the best range of agreement with a rate of difference lower than 10% (Table 9).

Regarding model validation, kappa index of agreement (K_IA_) comparison was made between the actual and simulated LULC maps of 2017. The validation of the model or K_IA_ statistics (Table 8) and the actual and predicted LULC change of the 2017 period (Table 10) result showed a good similarity between the actual and predicted maps of 2017. The overall kappa value is 87.3% which represents a strong agreement between the two map categories. Such a validation process was evaluating the agreement of the two maps (predicted and actual) in terms of the number of pixels in each LULU class and in term of their location of the pixels.

#### 3.3.2. Predicted Land/Use Land Cover

**Figure 7:**
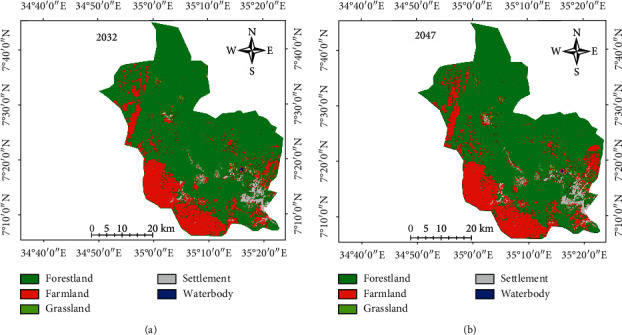
The 2032 and 2047 predicted LULC of MFBR.

The projected land use/land cover types of 2032 and 2047 were computed using the CA-Markov model as presented in Figure 7, whilst their area are given in Table 11. The area of forestland and grassland decreased from 72.4 in 2032 to 66.5 in 2047 and 1.2 in 2032 to 0.8 in 2047, respectively. A continuous increase in farmland and settlement will be observed in 2032 (21.5%) to 2047 (26.3) and 2032 (4.9) to 2047 (6.4), respectively. On the other hand, waterbody will depict almost a constant percentage in 2032 (0.05%) to 2047 (0.05%), while it will be decreased in area coverage from 135 ha to 126 ha in 2032 and 2047, respectively. In addition, as compared to LULC 2017–2047 farmland and settlement increased by 8.9% and 3%, respectively, while forestland and grassland decreased by 11.3% and 0.6%, respectively. The expansion of farmland and settlement is expected to increase at the expense of forestland and grassland.

**Figure 8:**
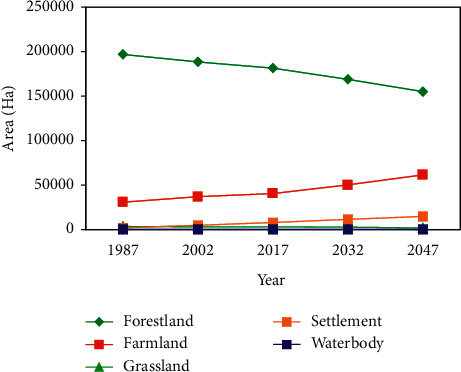
Trends of LULCC from 1985 to 2045 in MFBR.

In addition, as the result shows in Figure 8, a reduction of forestland cover from 84.4%, 80.78%, 77.8%, 72.4%, and 66.5%; grassland from 1.5%, 1.3%, 1.4%, 1.2%, and 0.8%; and waterbody from 0.06% to 0.05% were examined through the 1987–2047 study period, respectively. While farmland and settlement increased from13.2%, 15.82%, 17.4%, 21.5%, and 26.3% and 0.88%, 2.03%, 3.37%, 4.9%, and 6.4% at the expense of forestland through the 1987–2047 study period (Table 11).

The conversion of LU/LC classes also has been taken place between 2017 and 2045 predicted periods (Table 11). For example, between predicted periods 2017 and 2047 of forestland, grassland, and waterbody were reverted to farmland and settlement during the predicted periods by 26484.2 ha (11.3%), 1403.7 ha (0.6%), and 15 ha (0.01%), respectively. On the contrary, farmlands and settlements were expanded by 20922 ha (8.9%) and 6974 ha (3%) in the predicted periods, respectively.

### 3.4. Drivers of LULC Changes Based on Respondents View

Based on the respondents' survey results and field observation, multiple factors contributed to LULC changes in the study area. However, there was the difference in each of the factors to which the local community observed drivers of LULC changes. The survey and field observation showed that forestland and grassland were converted to farmland and settlements which are similar to LU/LC result. Regarding identification of driving factors of LULC changes, out of nine LULC change driving factors, the top four driving factors mentioned by the respondents in the study area were agriculture expansion (15.6%), human population growth (15.5%), wood extraction (14.6%), and risk of fire (14%), respectively (Table 12).

The population growth was perceived as the second driving factor causing LULC change. Based on 2007 Population Census of Ethiopia, the total population of Majang Zone was 59,248 [81], and the population was estimated to be 77,022 in 2014 and 82,268 in 2017. Thus, increment of the population between two census periods (2007 and 2014) varied from 3% in Akashi kebele to 27% in Ashani and Gonchi kebeles (Table 13). The increase of population in the study area demands land for agriculture, grazing land firewood, and settlement which could influence future land use/land cover.

Also, results from FGD and KII confirmed that population growth coupled with “resettlement of 1984/5” and “villagization of 2011” policies resulted in an extraexpansion of settlement and agricultural land at the expanses of forest and grassland. Besides, the “Land to Tiller” of 1970s policy including the absence of proper land use plans played a role in conversion of forest and grassland to settlement and farmland in the study area.

Based on the secondary data, the most recent phenomenon causing widespread forest destruction in the study area was an agricultural investment that began during 2003. About 19,165.83 ha of land were provided to investors or companies. For instance, Green Coffee, Verdanta, Marekose, and Afero-Tseyone companies were provided 6500 ha, 3012 ha, 3000 ha, and 2000 ha of land, respectively, in the study area (Table 14). It is inspected that land transfer to investors is a common phenomenon that aggravates land use/land cover changes. Also, the key informants reported that “land transfer to investors” puts strong pressure on the remaining forests to convert other LULC in the study area.

## 4. Discussion

### 4.1. Land Use/Land Cover Change

Computation of remotely sensed data is the well-established field of study that aids in articulating changes and patterns of land use/land covers in temporal and spatial aspects. Land use/land cover change analysis of the study area was run, and maps were generated for the last three decades, 1987–2017. The overall accuracies were attained by Landsat TM (85.4%), ETM+ (86%), and OLI-TIRS (87.3%) for the years 1987, 2002, and 2017 (Table 3). The values overall accuracy and kappa values above 80% indicate that the classification performance is satisfactory [82]. The results of this study are more or less related to other local studies such as Landsat TM (86.9%), ETM+ (85.8%), and OLI-TIRS (88.8%); Landsat MSS (83.1%), TM (85.8%), and ETM+ (88.7%); and Landsat MSS (85.5%), ETM+ (83.15%), and ETM+ (87.73%) [30, 41].

A total of five LULC types were identified in the study area in all study periods (1987–2017) (Figure 5); a forestland accounted for the largest proportion of farmland, grassland, settlement, and waterbody. Forestland and grassland were decreased and mainly transformed into farmland and settlement in all study periods (Table 6). This is possibly due to agricultural expansion as a result of human population increment in the study site. The finding of this research is consistent with other studies carried out in different parts of the country, for instance, Zeleke and Humi [21] in Dembecha area of northwest Ethiopia stated that 99% of the forest cover was transformed into farmland between 1957 and 1995. Similarly, Kindu et al. [8] in Munessa-Shashemene landscape of the Ethiopian highlands stated that almost 66.2% of woodland is converted to farmland. Many other local LULC studies also indicated similar trends [13, 29, 83, 84]. Also, a study in Baro river basin in southwestern Ethiopia showed the conversion of forest land to nonforestland between 1984 and 2010 mainly by the expansion of farmland and settlement [85]. In contrary, forestland was increased by 27% in Chemoga watershed in the Blue Nile, which was the result of the community afforestation program in a degraded hilly area in the watershed [86]. Moreover, land use/land cover conversions results revealed that a substantial loss and gains of LULC were inspected in the first and second study periods. For instance, forestland was converted to other LULC classes, while farmlands and settlements were gained from forestland and grassland during the first and second study periods (Tables 7 and 8). These changes may affect the habitat of key species in the area [87, 88]. Destruction of habitats and decrease in their sizes may lead to restriction and decline of species ecological niches.

### 4.2. Future Land Use/Land Cover Change

The CA-Markov chain is a stochastic procedures model that pronounces the possibility of change from one land use/land cover class into another land use/land cover class using a transition probability matrix [89, 90]. The most suitable model for predicting LULC change is the CA-Markov chain model [66]. This model is appropriate if the changes and procedures in a given landscape are challenging to describe. Regarding to model validation of this study, kappa index of agreement (KIA) comparison was made between the actual and simulated LULC map of 2017. The validation of the model or KIA statistics (Table 9) result showed a good similarity between the actual and predicted map of 2017. The actual and simulated maps for year 2017 were depicted reasonably similar for waterbody, while for the other LULC classes, there were almost slight differences between simulated and actual maps. However, considering the overall K_IA_ was a high level of agreement standards, and they were between 75% ≤kappa <100 [60, 70], which indicates the good agreement between the actual and simulated LULC maps [13, 48, 57, 71]. Therefore, the CA-Markov model is an effective tool and is reliable to simulate, predict, and analyse different changes of LULC in 2032 and 2047. With comparable kinds of agreement and disagreement result, [91] showed that the CA-Markov model might be taken as an effective model in prediction of LULC changes. The prediction of 2032 and 2047 result showed reduction of forestland, grassland, and waterbody cover, while farmland and settlement were increased at the expense of forestland and grassland in the predicted period (Figure 8), which is consistence with other findings [13, 17, 92]. The area extent of the forestland cover is likely to be taken over by farmland and settlement in the predicted period (Table 10), which may be due to future anthropogenic activities (farmland and settlement expansion) coupled with population increment in the area. These prediction results might be used as a guide for conservation planning in the area, assisting decision-makers to improve land use management plans to balance development and conservation.

### 4.3. Drivers of Land Use/Land Cover Changes

In general, LULC change is the result of the comprehensive influences of so many complex and various factors [93, 94]. Previous studies have revealed that on a global scale, human-driven land use/land cover change for most of the changes of land surface. However, the key driving factors vary according to the nature and magnitude of the area [95]. In this study, we analysed multiple driving factors contributed to LULC changes in the study area. Accordingly, the most important drivers of LULC change in the study area were agriculture expansion, population growth, wood extraction, and charcoal and fire (Table 12). Most importantly, agricultural expansion and population growth were more experienced as compared to other driving factors. This is similar with other finding reports in Ethiopia [13, 27, 35, 36]. Moreover, the driver plays a role in intensive loss of forestland to other land uses in the study area due to brutal drought and famine that affects the country, and the government implemented resettlement and villagization in the study area to combat the impacts of drought, which was aimed to move farmers from northern into southwestern parts of the country [96]. For instance, during the 1980s, at Gambella region (Godere, Zuria and Abobo) were settled about 11,234 households with land 15,600 ha, respectively [97]. FGD and KII also confirmed that population growth coupled with resettlement (1984) and villagization (2010) in the study area that resulted in further expansion of agricultural land as expanses of forestland which was the main driving force of LULC change in the area. For instance, increased population number is observed in the sample kebele both in national count in 2007 and 2014 which was evident largely through the expansion of farmlands at the expense of forestland cover [98, 99]. Likewise, a significant loss of the grasslands to other land use was observed in the area as a result of the expansion of agriculture and rapid population growth. Similar results were reported in different parts of Ethiopia [13, 83, 96, 100].

## 5. Conclusion

Land use/land cover change (LULC) analyses are crucial for a well-informed decision-making regarding proper land uses planning policy. This study identified five LULC classes such as forestland, farmland, grassland, settlement, and waterbody in the study area, for the study periods of 1987–2017. Based on the LULC changes, farmland and settlement increased while forestland and grassland were reduced in the study period of time (1987–2017). A broad-spectrum trend was inspected as increment of farmland and settlement areas; meanwhile, shrinkage of forestland, grassland, and waterbody will continue in the near future, 2032–2047. Eventually, based on the respondents ranking, the main drivers of LULC changes were identified as agricultural expansion, human population growth, shifting cultivation, fuel wood extraction, and fire risk. Moreover, results from focus group discussion (FGD) also confirmed that population growth coupled with resettlement and villagization have resulted in further expansion of agricultural land as expanses of forestland. Generally, substantial LULC changes were observed and most likely continued onward until the specified future period of this study. Hence, a rational land use plan should be proposed in order to sustain livelihoods of local communities, resources of MFBR, and the environment. Likewise, the predicted model applied in this study delivers basic information that the planner should consider extensive driving factors of physical, social, and economic associated with the complex use of land. Moreover, this study also proposes a further study on the impacts brought by LULC change, specifically climate and watershed hydrology; meanwhile, this study addressed only LULC change, driving forces behind the changes, and future prediction.

## Figures and Tables

**Table 1:** Satellite images used for LULC change analysis and their characteristics.

Satellite image	Path/Row	Sensor	Resolution (m)	No. of bands	Date of acquisition	Cloud cover
Landsat-5	170/55	TM	30	7	01/22/1987	0
	171/55	TM	30	7	01/31/1987	0

Landsat-7	170/55	ETM+	30	8	12/28/2002	0
	171/55	ETM+	30	8	12/19/2002	0

Landsat-8	170/55	OLI-TIRS	30	11	01/08/2017	0
	171/55	OLI-TIRS	30	11	01/15/2017	0

**Table 2:** Description of the LULC classification system.

LULC	Description
Forestland	Land covered with trees reaching 5 m in height, 0.5 ha in area, and a canopy cover of >10%.
Farmland	Areas covered with annual and perennial crops
Settlement	These areas both in urban and small rural residential places including trees in individual garden and big and small size roads
Grassland	Areas dominantly covered with grasses and shrubs
Waterbody	Waterbody: area which holds water (lakes), rivers, and marshy land

Source: [45].

**Table 3:** Accuracy assessment of 1987, 2002, and 2017 classified images.

Land use/land cover	1987		2002		2017	
	Producer accuracy	User accuracy	Producer accuracy	User accuracy	Producer accuracy	User accuracy
Forestland	86.3	92	87.8	90.2	89	91.9
Farmland	86.7	85.68	89.33	82.2	90	85.78
Grassland	71.6	84.5	75	80.9	80.5	79.9
Settlement	80.7	89	81.6	85.9	83.4	81.7
Waterbody	79.6	81	84.4	83.8	85.7	82
Overall accuracy (%)	85.4		86		87.3	
Kappa coefficient	0.81		0.82		0.83	

**Table 4:** The number of participants in the HH, FGD, and KII in all kebeles.

Kebele	Total population	Total HH	Selected HH	Selected FGD	Selected KII
Ashani^1^	1,758	464	22	8	3
Baya^1^	1,565	422	20	8	3
Fejeji^1^	1,721	469	22	8	3
Newe^1^	1,358	360	17	8	3
Akashi^2^	6,170	1655	77	8	3
Dunchaye^2^	2,554	685	32	8	3
Gonchi^2^	1,556	428	20	8	3
Gelesha^2^	2,376	651	30	8	3
Total	19,561	5,134	240	64	24

*Note.* ^1^Mengeshi and ^2^Godere districts.

**Table 5:** Area of LULC class from 1987 to 2017 periods in MFBR.

Land use/land cover	1987		2002		2017	
	Area (ha)	Area (%)	Area (ha)	Area (%)	Area (ha)	Area (%)
Forestland	196761.6	84.4	188413.7	80.8	181504.9	77.8
Farmland	30781.8	13.2	36906.4	15.8	40554.8	17.4
Grassland	3509.2	1.5	3079.6	1.3	3192.2	1.4
Settlement	2050.7	0.9	4744.3	2.0	7866.2	3.4
Waterbody	141.0	0.06	141.0	0.06	141.0	0.06
Total	233254	100%	233254	100%	233254	100%

**Table 6:** Percentage and rate of changes occurred in MFBR from 1987 to 2017 periods.

LU/LC	PC			RC (ha/year)		
	1987–2002	2002–2017	1987–2017	1987–2002	2002–2017	1987–2017
Forestland	−4.4	−3.8	−8.4	−556.5	−460.6	−1017.1
Farmland	16.6	9.0	24.1	408.3	243.2	651.5
Grassland	−13.6	3.6	−9.5	−28.6	7.5	−21.1
Settlement	56.7	39.8	73.9	179.6	208.1	387.7
Waterbody	0.0	0.0	0.0	0.0	−0.3	−0.3

*Note.* MFBR = Majang Forest Biosphere Reserve, PC = percentage of change, and RC = rate of change.

**Table 7:** Transition area matrix (ha) between 1987 and 2002 in MFBR.

1987	2002					
	Forestland	Farmland	Grassland	Settlement	Waterbody	Total
Forestland	182378	3732	1800	458	3	188372
Farmland	9093	26486	786	519	0	36884
Grassland	2502	47	520	10	0	3079
Settlement	2764	510	402	1062	0	4739
Waterbody	3	0	0	0	138	141
Total	196740	30775	3509	2049	141	233254

**Table 8:** Transition area matrix (ha) between 2002 and 2017 in MFBR.

2002	2017					
	Forestland	Farmland	Grassland	Settlement	Waterbody	Total
Forestland	174287	4312	1773	1114	6	181492
Farmland	8384	29956	829	1375	0	40544
Grassland	2237	206	455	293	0	3191
Settlement	3467	2418	21	1958	0	7864
Waterbody	2	0	0	0	134	136
Total	188377	36892	3078	4740	140	233254

**Table 9:** Statistical validation of the CA-Markov chain model.

Statistics	Value (%)
K_standard_	85.3
K_no_	81.2
K_location_	81.4
K_locationstrata_	80.8
Overall K	87.3

**Table 10:** Comparison of actual and simulated LULC changes in 2017.

LULC class	Actual (2017)		Simulated (2017)		Changes	
	Area (ha)	Area (%)	Area (ha)	Area (%)	RC (ha)	PC
Forestland	181504.9	77.8	180980.7	77.6	−524.2	−0.2
Farmland	40554.8	17.4	41014.7	17.5	459.9	0.2
Grassland	3192.2	1.4	3002.5	1.3	−189.7	−0.1
Settlement	7866.2	3.4	8120.6	3.5	254.4	0.1
Waterbody	141	0.06	141	0.06	−1	0
Total	233254	100%	233254	100		

*Note.* PC = percentage of change, RC = rate of change.

**Table 11:** LULC area change (ha) from 2017 to 2047of MFBR.

LULC class	2017		2032		2047		(2017–2047)	
	Area	%	Area	%	Area	%	RC (ha)	PC
Forestland	181504.9	77.8	168800.7	72.4	155020.7	66.5	−26484.2	11.3
Farmland	40554.8	17.4	50151.8	21.5	61476.8	26.3	20922	8.9
Grassland	3192.2	1.4	2740.2	1.2	1788.5	0.8	−1403.7	0.6
Settlement	7866.2	3.4	11426.2	4.9	14840.2	6.4	6974	3
Waterbody	141	0.06	135	0.05	126	0.05	−15	0.01
Total	233254	100%	233254	100%	233254	100%		

*Note.* MFBR = Majang Forest Biosphere Reserve, PC = percentage of change, and RC = rate of change.

**Table 12:** Land use/land cover change key drivers and ranking.

Drivers of LULC changes	Percentage (%)	Rank
Agriculture expansion	15.6	1
Population growth	15.5	2
Wood extraction (for charcoal, fuel wood, and construction)	14.6	3
Fire	14.0	4
Expansion of settlement	11.6	5
Infrastructure development	10.6	6
Lack of forest policies and laws	7.4	7
Limited capacities of the forest sector	6.4	8
Absence of land use planning	4.3	9

**Table 13:** Population number and growth rate in the sample kebeles of Majang Zone.

Name of kebele	2007	2014 (E)	Growth between 2007 and 2014	Change between 2007 and 2014 (%)	Growth rate (%) between 2007 and 2014	Doubling time after 2014
Ashani^1^	388	1758	1370	353	27	3
Baya^1^	381	1565	1184	311	24	3
Fejeji^1^	446	1721	1275	286	22	3
Newe^1^	307	1358	1051	342	26	3
Akashi^2^	4,269	6170	1901	45	3	20
Dunchaye^2^	1,536	2554	1018	66	5	14
Gelesha^2^	1,249	2376	1127	90	7	10
Gonchi^2^	345	1556	1211	351	27	3

*Note.* ^1^Mengeshi and ^2^Godere district. E, estimated.

**Table 14:** Leased land for private companies investment in Majang Zone.

Company name	Area (ha)	Year of licensed	Lease period (years)
Green Coffee	6500	2015	50
Verdanta	3012	2015	50
Marekose	3000	2015	50
Afero-Tseyone	2000	2015	50
G/Medihen	1400	2014	50
Majang Agro-Industry	1000	2015	50
Shake Agro-Industry	763	2015	50
Ebdayetaye	488	2013	50
Tekalign	355	2015	50
Siraje Negaw	362	2003	50
Adenew Angelo	189.83	2006	50
Total	19,165.83		

## Data Availability

The data used to support the findings of this study are included within this article without restriction and in its supporting information file.

## Supplementary Materials

Supplementary Materials S1 Table. Attributes of sampled households in the study area (*N* = 240), exploring overviews of respondent profiles, specifically socioeconomic attributes such as family size, occupation, size of landholdings, and education status are essential in terms of identifying and understanding driving factors of land use/land cover changes in the study area. Henceforth, some of the relevant attributes are summarized in this supporting table. S2 Table. Mean rainfall and temperature in the study area station which is used in suitability map preparation to predict the future LULC (MAR, mean annual rainfall; MAT, mean annual temperature). S3 and S4 Tables. Mean monthly mean temperature and rainfall (30 year mean) for climate description in Tinishu Meti and Tinishu Ermichi.
