# Supplementary material for: Analysis of the Current and Future Prediction of Land Use/Land Cover Change Using Remote Sensing and the CA-Markov Model in Majang Forest Biosphere Reserves of Gambella, Southwestern Ethiopia
Source: ScientificWorldJournal. 2021 Feb 23;2021:6685045. doi: 10.1155/2021/6685045 (PMC7925022; doi:10.1155/2021/6685045)
Supplement: Supplementary Materials — S1 Table. Attributes of sampled households in the study area (N = 240), exploring overviews of respondent profiles, specifically socioeconomic attributes such as family size, occupation, size of landholdings, and education status are essential in terms of identifying and understanding driving factors of land use/land cover changes in the study area. Henceforth, some of the relevant attributes are summarized in this supporting table. S2 Table. Mean rainfall and temperature in the study area station which is used in suitability map preparation to predict the future LULC (MAR, mean annual rainfall; MAT, mean annual temperature). S3 and S4 Tables. Mean monthly mean temperature and rainfall (30 year mean) for climate description in Tinishu Meti and Tinishu Ermichi. [file 6685045.f1.docx]

**S1 Table Attributes of sampled households in the study area (N = 240)**

| **No** | **Household attributes** | **Value** |
| --- | --- | --- |
| 1 | Interview gender (male, %) | 80 |
| 2 | Average Household age (year) | 45.54 |
| 3 | Level of Education (Literate, %) | 30 |
| 4 | Household occupation (farming, %) | 95 |
| 5 | Average HH size (number) | 6.03 |
| 6 | Average land holding size (ha) | 4.23 |

**S2 Table Mean Rainfall and Temperature in the study area station which is used in suitability map preparation to predict the future LULC (MAR= mean annual rainfall, MAT= mean annual temperature**

| **Year** | **MAR** | **MAT** |
| --- | --- | --- |
| 1987 | 73.4 | 11.3 |
| 1988 | 186.3 | 11.5 |
| 1989 | 104.5 | 11.8 |
| 1990 | 107.8 | 12.1 |
| 1991 | 136.8 | 12.4 |
| 1992 | 101.3 | 12.5 |
| 1993 | 118.9 | 12.6 |
| 1994 | 149.1 | 12.9 |
| 1995 | 154.5 | 13.3 |
| 1996 | 168.6 | 13.6 |
| 1997 | 146.3 | 13.9 |
| 1998 | 166.1 | 14.3 |
| 1999 | 142.7 | 14.6 |
| 2000 | 144.1 | 14.9 |
| 2001 | 140.7 | 14.8 |
| 2002 | 103.7 | 14.1 |
| 2003 | 275.7 | 13.3 |
| 2004 | 235.9 | 15.2 |
| 2005 | 188.9 | 15.5 |
| 2006 | 172.9 | 15.7 |
| 2007 | 149.5 | 15.9 |
| 2008 | 149.9 | 16.3 |
| 2009 | 109.5 | 15.4 |
| 2010 | 128.9 | 16.5 |
| 2011 | 159.5 | 16.8 |
| 2012 | 117.9 | 16.8 |
| 2013 | 161.5 | 17.2 |
| 2014 | 114.1 | 17.4 |
| 2015 | 153.9 | 16.5 |
| 2016 | 172.5 | 17.6 |
| 2017 | 188.9 | 17.9 |

**S3: Mean monthly mean Temperature and rainfall (30 year mean) for climate description Teneshu Metti**

| **Months** | **Teneshu Metti** | | |
| --- | --- | --- | --- |
|  | **Rainfall** | **Temp Max** | **Temp Min** |
| **January** | 41.68 | 29.83 | 13.93 |
| **February** | 38.9467 | 31.81 | 14.92 |
| **March** | 98.7333 | 31.49 | 15.45 |
| **April** | 176.797 | 30.4 | 16.12 |
| **May** | 222.323 | 29.29 | 16.04 |
| **June** | 211.683 | 28.25 | 15.08 |
| **July** | 212.847 | 27.49 | 14.81 |
| **August** | 188.963 | 27.65 | 14.23 |
| **September** | 210.427 | 28.69 | 14.24 |
| **October** | 166.323 | 28.95 | 15.05 |
| **November** | 128.02 | 29.54 | 14.15 |
| **December** | 77.3733 | 29.43 | 14.62 |

**S4: Mean monthly mean Temperature and rainfall (30 year mean) for climate description Teneshu Ermichi**

| **Months** | **Ermichi** | | |
| --- | --- | --- | --- |
|  | Rainfall | Temp Max | Temp Min |
| **January** | 51.8 | 27.83 | 11.93 |
| **February** | 46.9 | 29.81 | 12.92 |
| **March** | 116.1 | 29.49 | 13.45 |
| **April** | 214.5 | 28.4 | 14.12 |
| **May** | 261.0 | 27.29 | 14.04 |
| **June** | 230.8 | 26.25 | 13.08 |
| **July** | 238.1 | 25.49 | 12.81 |
| **August** | 250.3 | 25.65 | 12.23 |
| **September** | 240.6 | 26.69 | 12.24 |
| **October** | 218.3 | 26.95 | 13.05 |
| **November** | 142.6 | 27.54 | 12.15 |
| **December** | 77.0 | 27.43 | 12.62 |
